# Longitudinal Changes in Insulin Resistance in Normal Weight, Overweight and Obese Individuals

**DOI:** 10.3390/jcm8050623

**Published:** 2019-05-08

**Authors:** Alice Tang, Adelle C. F. Coster, Katherine T. Tonks, Leonie K. Heilbronn, Nicholas Pocock, Louise Purtell, Matthew Govendir, Jackson Blythe, Jialiang Zhang, Aimin Xu, Donald J. Chisholm, Nathan A. Johnson, Jerry R. Greenfield, Dorit Samocha-Bonet

**Affiliations:** 1 Diabetes and Metabolism Division, Garvan Institute of Medical Research, Sydney 2010, Australia; a.tang@unswalumni.com (A.T.); k.tonks@garvan.org.au (K.T.T.); louise.purtell@qut.edu.au (L.P.); m.govendir@unsw.edu.au (M.G.); jackson.blythe@outlook.com (J.B.); d.chisholm@garvan.org.au (D.J.C.); 2 St. Vincent’s Clinical School, UNSW Sydney, Sydney 2010, Australia; n.pocock@unsw.edu.au; 3 School of Mathematics and Statistics, UNSW Sydney, Sydney 2052, Australia; A.Coster@unsw.edu.au; 4 Department of Endocrinology and Diabetes Center, St. Vincent’s Hospital, Sydney 2010, Australia; 5 Adelaide Medical School, University of Adelaide, Adelaide 5005, Australia; leonie.heilbronn@adelaide.edu.au; 6 State Key Laboratory of Pharmaceutical Biotechnology, The University of Hong Kong, Hong Kong, China; hjzhang@hku.hk (J.Z.); amxu@hku.hk (A.X.); 7 Faculty of Health Sciences, The University of Sydney, Camperdown 2006, Australia; nathan.johnson@sydney.edu.au

**Keywords:** insulin resistance, obesity, fat-free mass, hyperinsulinaemic-euglycaemic clamp, liver fat

## Abstract

Background: Large cohort longitudinal studies have almost unanimously concluded that metabolic health in obesity is a transient phenomenon, diminishing in older age. We aimed to assess the fate of insulin sensitivity per se over time in overweight and obese individuals. Methods: Individuals studied using the hyperinsulinaemic-euglycaemic clamp at the Garvan Institute of Medical Research from 2008 to 2010 (*n* = 99) were retrospectively grouped into Lean (body mass index (BMI) < 25 kg/m^2^) or overweight/obese (BMI ≥ 25 kg/m^2^), with the latter further divided into insulin-sensitive (Ob_Sen_) or insulin-resistant (Ob_Res_), based on median clamp M-value (M/I, separate cut-offs for men and women). Fifty-seven individuals participated in a follow-up study after 5.4 ± 0.1 years. Hyperinsulinaemic-euglycaemic clamp, dual-energy X-ray absorptiometry and circulating cardiovascular markers were measured again at follow-up, using the same protocols used at baseline. Liver fat was measured using computed tomography at baseline and proton magnetic resonance spectroscopy at follow-up with established cut-offs applied for defining fatty liver. Results: In the whole cohort, M/I did not change over time (*p* = 0.40); it remained significantly higher at follow-up in Ob_Sen_ compared with Ob_Res_ (*p* = 0.02), and was not different between Ob_Sen_ and Lean (*p* = 0.41). While BMI did not change over time (*p* = 0.24), android and visceral fat increased significantly in this cohort (*p*_time_ ≤ 0.0013), driven by Ob_Res_ (*p* = 0.0087 and *p* = 0.0001, respectively). Similarly, systolic blood pressure increased significantly over time (p_time_ = 0.0003) driven by Ob_Res_ (*p* = 0.0039). The best correlate of follow-up M/I was baseline M/I (Spearman’s *r* = 0.76, *p* = 1.1 × 10^−7^). Conclusions: The similarity in insulin sensitivity between the Ob_Sen_ and the Lean groups at baseline persisted over time. Insulin resistance in overweight and obese individuals predisposed to further metabolic deterioration over time.

## 1. Introduction

The obesity rate has nearly tripled worldwide since 1975, with World Health Organization data indicating that in 2016, 39% of adults were overweight and 13% were obese [1]. In the United States, recent figures indicate that 40% of adults are obese, with the obesity rate in the 40–60 year old age group exceeding 40% [1].

While obesity is a risk factor for metabolic disease, sub cohorts with obesity not complicated by the metabolic syndrome have been described. These so called “metabolically healthy obese” may have reduced risk of type 2 diabetes, cardiovascular disease and all-cause mortality compared with individuals with obesity and the metabolic syndrome [2]. The criteria used to define metabolically healthy obesity (MHO) varies widely across studies. With at least 30 different definitions applied, the prevalence of MHO ranged from 6 to 75% in the obese population [3]. Most studies included blood pressure, high density lipoprotein (HDL) cholesterol, fasting plasma glucose and/or triglyceride in their definition. Less than half included the insulin resistance surrogate homeostatic model assessment of insulin resistance (HOMA-IR), and less than one-third included diabetes [3]. Compared to the use of the metabolic syndrome criteria to sub-categorise obesity, fewer studies have used insulin sensitivity per se (that is, sub-categorisation into insulin-sensitive obesity (Ob_Sen_) and insulin-resistant obesity (Ob_Res_)). Most studies considering insulin resistance used the insulin resistance surrogate homeostatic model assessment of insulin resistance (HOMA-IR) [3]; some smaller cohort studies used the gold-standard hyperinsulinaemic-euglycaemic clamp [4,5].

Despite having a better metabolic health profile compared to the metabolically unhealthy obesity group, longitudinal studies with diabetes and cardiovascular disease endpoints reported that MHO individuals held an intermediate health status, such that they were still worse off than the healthy normal weight individuals [6]. Moreover, recent longitudinal studies have shown that a large proportion of MHO progress to the unhealthy category over time [7,8,9,10,11,12,13,14]. Predictors of loss of metabolic health in MHO include older age [9,15] and poorer cardio-metabolic risk at baseline, including lower HDL [9,11,12,13], higher triglycerides [11,12,13], higher central adiposity [11,15] and insulin resistance [9,11,12,13].

While there have been studies evaluating the stability of the MHO phenotype over time, to our knowledge no study has reported the durability of insulin-sensitivity per se, measured by the gold-standard hyperinsulinaemic-euglycaemic clamp in overweight and obese individuals. Focusing on the change in insulin resistance over time is key to dissecting the mechanism underlying the development of cardio-metabolic disease in individuals at risk. In the present study, we aimed to trace the change in insulin resistance, and to uncover predictors of insulin resistance in older age. The secondary aims were to trace the change in body composition, fat distribution and metabolic markers over time in a well-phenotyped cohort studied approximately 5 years apart.

## 2. Experimental Section

### 2.1. Participants

The Insulin Sensitivity in Obesity Study (ISOS) was approved by the St Vincent’s Hospital Human Research Ethics Committee and the Garvan Institute of Medical Research Governance Office and registered at ClinicalTrials.gov (NCT02017210).

**Figure 1:**
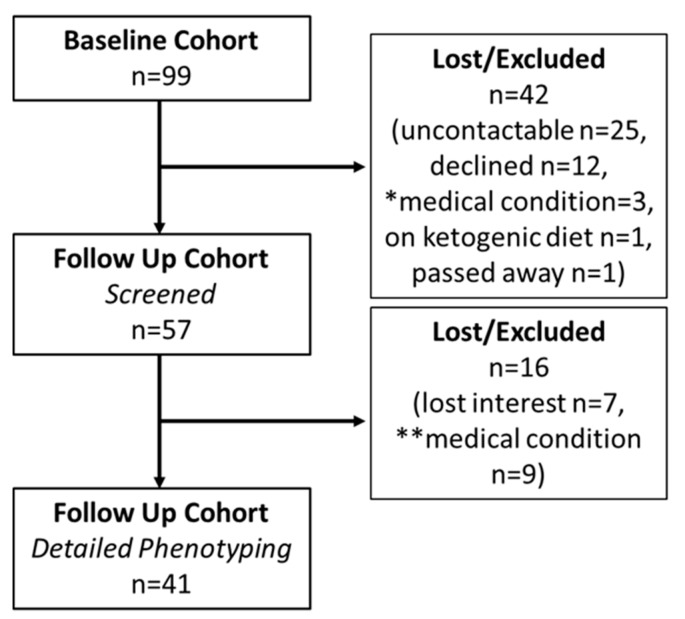
Study Flow (Consort Diagram). * Medical conditions precluding from follow-up screening included bowel cancer, mitral valve repair and cardiac arrhythmia, chronic lymphocytic lymphoma, breast cancer on letrozole, non-Hodgkin’s lymphoma, sleeve gastrectomy and trying to conceive. ** Medical conditions precluding from detailed phenotyping included tetralogy of fallot, venous thrombotic disease, venous access difficulty (axillary lymph node clearance), iron deficiency anemia of uncertain cause, significant coronary artery disease (requiring stenting, coronary artery bypass grafting and aspirin therapy), immunosuppressant therapy for psoriatic arthritis, renal failure and significant hypertension (189/109 mmHg), severe untreated autoimmune urticaria, excessive alcohol consumption (>20 g/day for a female participant) with paroxysmal atrial fibrillation.

Participants of two previous separate studies performed at the Garvan Institute of Medical Research (Darlinghurst, NSW, Australia) [16,17] were contacted by postal mail to gauge interest in participating in a follow-up study. At the time of these previous studies (“baseline studies”), participants agreed to be contacted in the future for other potential studies, but were not contacted during the gap between the baseline and follow-up studies. The data collected in the original studies, performed between 2008 and 2010, formed the baseline data. In the original studies, exclusion criteria included weight change greater than 2 kg in the preceding 6 months, self-reported regular exercise for periods longer than 60 min/week, treatment with medications known to affect insulin sensitivity or carbohydrate metabolism, known renal, cardiac, or liver disease and current cancer, women planning pregnancy, consumption of more than 20 and 40 g/day alcohol for women and men, respectively, and smoking >10 cigarettes/day. Type 2 diabetes was defined by medical history or by fasting blood glucose (≥7.0 mmol/L) and/or 2-h blood glucose (≥11.1 mmol/L) following a 75 g oral glucose tolerance test (OGTT), which was performed during screening [17]. Non-diabetic participants from the baseline cohort (*n* = 99, Figure 1 (Consort Diagram)) had their interest for participating in a follow-up study gauged by postal mail. Four were of Asian descent, the rest were Caucasian. Of the baseline participants, 25 were uncontactable or living interstate, 12 declined, 3 had a medical condition precluding participation in the follow-up study, 1 was actively losing weight, and 1 had passed away (Figure 1). Fifty-seven individuals were invited to the Clinical Research Facility for a screening visit to assess their medical status, and, if willing, progress to an additional study visit, which included detailed phenotyping, as described below. Sixteen individuals who were screened at follow-up did not progress to the detailed phenotyping study, 7 as they had lost interest and 9 due to medical conditions (Figure 1).

### 2.2. Baseline and Follow-Up Studies

Protocols applied during the baseline and follow-up studies were almost identical, as detailed below.

#### 2.2.1. Screening Visit

Participants were invited to the Clinical Research Facility to a screening visit in the morning after an overnight fast. The study included physical examination, weight, height, waist and hip circumference and blood pressure (Omron, Model IA1B, Port Melbourne, Australia) measurements. Fasting blood samples were collected. Physical activity was assessed using the Stanford 7-day activity questionnaires, as described previously [18].

#### 2.2.2. Detailed Phenotyping

On a separate day, participants underwent hyperinsulinaemic-euglycaemic clamp and dual-energy X-ray absorptiometry (DXA) to assess insulin resistance and body composition, respectively. Liver fat was assessed by computed tomography (CT) at baseline and, to minimise cumulative radiation exposure, proton magnetic resonance spectroscopy (^1^H-MRS) at follow-up, as detailed below. These are available for the entire baseline cohort (*n* = 99) and for a sub-cohort of the follow-up cohort (*n* = 41, Figure 1).

Participants were instructed to abstain from physical activity and alcohol for 2 days prior to the study day and to arrive to the Clinical Research Facility after an overnight fast. Hyperinsulinaemic-euglycaemic clamp was performed using the same protocols used at the baseline studies [16,17]. Intravenous access was obtained by cannulating each arm at the ante-cubital fossa. One arm was used for obtaining venous blood glucose for monitoring throughout the clamp. The contralateral arm was used for insulin (Actrapid, Novo Nordisk Pharmaceuticals, Baulkham Hills, NSW, Australia) and 25% glucose (Baxter Healthcare, Toongabbie, NSW, Australia) infusion. Insulin was infused at a constant rate, with a supra-physiological concentration chosen to suppress hepatic gluconeogenesis, so that differences in net glucose disposal between groups could be interpreted as differences in peripheral (mainly muscle) glucose disposal. Insulin infusion rate was the same as that used in the baseline studies, 60 [16] or 80 [17] mU/m^2^/min. Glucose was infused at a variable rate to maintain a venous glucose concentration of 4.5–5.5 mmol/L. Insulin resistance (median clamp M-value (M/I)) was expressed in terms of average glucose infusion rate (GIR) obtained from the last 30 min of the clamp (steady state) normalised to fat-free mass (FFM) and steady state serum insulin concentrations (I).

DXA (Lunar Prodigy, GE Healthcare, Chicago, IL, USA) was used to measure total body fat mass and FFM (enCORE software Version 16), the android and gynoid region, and visceral fat (CoreScan software, GE Healthcare) at St Vincent’s Clinic Bone Densitometry (Sydney, Australia). Standard fields of view automatically placed by the DXA software included the android (abdominal) and gynoid (hip) regions. The lower margin of the android region was defined by the pelvic cut line and the lateral margins by the arm cut lines. The upper margin was set at a height that is equal to 20% of the distance between the pelvic cut line and the neck cut line. The height of the android region is used to delineate the gynoid area. The upper border is set below the pelvic cut at a distance that is 1.5 times the height of the android region. The height of the gynoid region is twice the height of the android region. The lateral borders are defined by the outer leg cut lines (GE Healthcare Lunar enCORE-based X-ray Bone Densitometer User Manual, 2013). Visceral adipose tissue was determined from the android region by the software algorithm, which corrects total fat mass in the android region for estimated subcutaneous fat, providing visceral adipose tissue volume in cm^3^ (which may be converted to mass using the multiplicative constant 0.94 g/cm^3^).

Liver fat was measured by liver density, an inverse measure of fat content, derived from CT (Gemini GXL, Philips, The Netherlands) during the baseline study. Briefly, liver attenuation values (measured in Hounsfield units [Hu]) from a 10 mm axial slice at T12/L1 level were derived. Three regions of interest were measured and averaged (one in the left lobe, two in the right lobe) to calculate the liver attenuation value. ^1^H-MRS was performed at the follow-up studies, using a 1.5-Tesla Ingenia whole-body system (Philips Medical Systems, Best, The Netherlands). Briefly, image-guided, localised ^1^H-MRS were acquired from a voxel of 3.0 × 3.0 × 3.0 cm, with volumes of interest centred within the right lobe of the liver. Subjects lay supine, with spectra acquired using the PRESS (point resolved spectroscopy) technique (TR = 2800 ms, TE = 36 ms, six measurements, 1024 sample points) during respiratory gating. Spectral data were post-processed by magnetic resonance user interface software (jMRUI version 3.0, EU Project) as described [19]. In order to compare the liver fat content gathered in the baseline and follow-up studies using different imaging methods (CT and MRS, respectively), we considered the imaging modalities’ correlations with the gold-standard method for defining fatty liver, the histological grading of liver tissue. Normal liver fat content is most commonly defined as macroscopic steatosis in less than 5% of hepatocytes [20]. To define fatty liver, we applied cut-offs derived in the study by Van Werven et al., comparing CT, ^1^H-MRS and liver histology in a similar cohort. In that study of 46 men and women (average body mass index (BMI) 27 kg/m^2^, 59 years of age) fatty liver (>5% liver fat by histology) correlated to a CT measure of 54.2 Hu (with 74% sensitivity and 70% specificity) and ^1^H-MRS measure of 1.8% (with 91% sensitivity and 87% specificity) [21]. Hence, the presence of excess liver fat accumulation was determined by a liver attenuation value of less than 54.2 Hu at baseline and a liver fat content of greater than 1.8% at follow-up.

### 2.3. Biochemical Analyses

Whole blood glucose was analysed using a glucose analyser (YSI 2300 Stat Plus, YSI Incorporated Ohio USA, Yellow Springs, OH, USA), and serum insulin was measured using radioimmunoassay (Human Insulin-Specific RIA, Millipore, Burlington, MA, USA). Serum lipid profile was analysed by a spectrophotometric assay (Advia® 2400 Chemistry System [Siemens Medical Solutions Diagnostics, Tarrytown, NY, USA]), with low-density lipoprotein (LDL) calculated using the Friedewald equation. Fibroblast growth factor (FGF)-19, FGF-21, total adiponectin, fatty acid-binding protein 4 (FABP4), lipocalin 2 and retinol-binding protein 4 (RBP4) were measured by ELISA (Antibody and Immunoassay service, University of Hong Kong), as previously described [22]. Serum lipid profile and the circulating adipokines were measured in serum samples, from the baseline and follow-up study stored at −80 °C, after the completion of the follow-up study, whereas whole blood glucose was measured in fresh samples when collected, on the baseline and follow-up studies. Serum insulin concentrations were measured in samples batched and stored at −80 °C until analysis, at the end of the baseline and follow-up studies, separately.

### 2.4. Statistical Analysis

Groupings of the cohort were made using the BMI and M/I value from the baseline data. The Lean group included all individuals with BMI < 25 kg/m^2^. Those with BMI ≥ 25 kg/m^2^ were categorised as Ob_Res_ if their M/I were below the median M/I value for each gender and Ob_Sen_ if above.

The baseline and follow-up data were analysed to check for homoscedasticity using a Bartlett test [23]. To compare the differences in outcome measures between groups, if the variances across the groups were equal, one-way ANOVA was performed followed by a Tukey–Kramer posthoc test for significance. In the case of unequal variances, Welsh’s ANOVA was calculated and the Games–Howell posthoc test [24] used to determine significant differences. In both cases, the significance cutoff was 0.05.

To analyse the changes in the variables as a function of time, the rate of change was determined as $\frac{\mathrm{Follow}-\mathrm{up}\;\mathrm{Value}\;-\;\mathrm{Baseline}\;\mathrm{Value}}{\mathrm{Time}\;\mathrm{between}\;\mathrm{measurements}}$ for each baseline group. To assess whether the variables for each group changed significantly over time, the values of the changes were tested to see if they were significantly different from zero. A one-sample *t*-test was performed both for all the data (*p*_time_) and for each group. The significance level in the latter case was corrected for multiple comparisons using a Bonferroni correction (significance at *p* ≤ 0.0167), where the significance cutoff of 0.05 was divided by 3, the number of groups. As the change with time data had unequal variances across the groups, Welsh’s ANOVA with the Games–Howell posthoc test was used to determine significant differences between the groups (*p*_group_). A Chi-squared test was performed to test the change in liver fat status in the different groups from baseline to follow-up.

Pairwise Spearman correlations between baseline and follow-up values were calculated. Both the correlations with *p* ≤ 0.05 and *p* ≤ 0.01, taking multiple comparisons into account, were determined.

Statistical analyses were implemented in MATLAB (R2018b The Mathworks, Inc. 2018, Natick, MA, USA).

## 3. Results

### 3.1. Baseline Characteristics

The cohort characteristics are described in Table 1. Median age in this cohort was 49.8 years (interquartile range (IQR) 22.9, (37.2, 60.0)). When retrospectively grouping the baseline cohort, Ob_Sen_ were significantly younger than Ob_Res_ (*p* = 0.001) and not different from the Lean group (*p* = 0.99). BMI in Ob_Res_ was marginally higher than Ob_Sen_ (*p* = 0.056). Fat-free mass and fat mass were not significantly different between Ob_Sen_ and Ob_Res_ (*p* ≥ 0.24). While fat content in the gynoid region was not different between the overweight/obese groups, fat content in the android region in Ob_Sen_ was intermediate between Lean and Ob_Res_, and a similar observation was noted for visceral adipose tissue volume (Table 1). Liver fat measured by CT attenuation index in Ob_Sen_ was intermediate between the higher level measured in Ob_Res_ and lower level measured in the Lean group (Table 1).

Blood pressure (systolic and diastolic) and fasting blood glucose measured in Ob_Sen_ were intermediate between that measured in Lean and Ob_Res_ (Table 1). Fasting serum triglycerides were significantly higher in both Ob_Sen_ and Ob_Res_ compared with Lean (*p* ≤ 0.002), while fasting HDL-cholesterol and LDL-cholesterol were not significantly different between the groups (*p* ≥ 0.15, Table 1).

**Figure 2:**
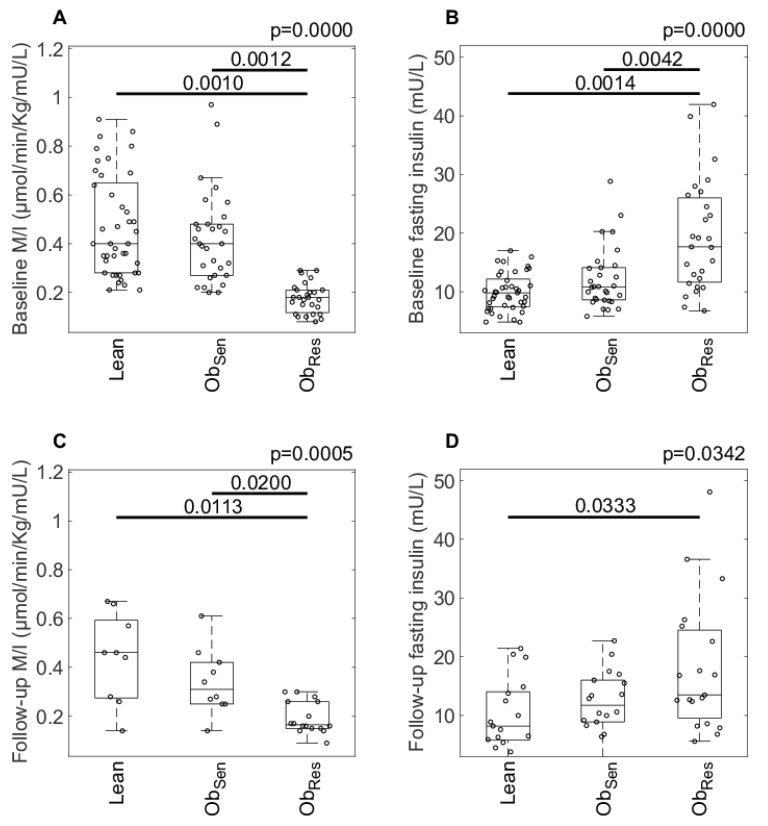
Insulin resistance at baseline and follow-up in Lean and overweight/obese insulin-resistant (Ob_Res_) and insulin-sensitive (Ob_Sen_) individuals. Insulin resistance (median clamp M-value (M/I), **A** and **C**) and fasting serum insulin (**B** and **D**) measured at baseline and follow-up, respectively, in Lean and overweight/obese individuals. Data are individual data points with median and interquartile range (IQR). Welsh’s ANOVA was calculated and the Games–Howell posthoc test was used to determine significant differences. The *p* for the Welsh’s ANOVA is indicated at the top of the plots, with the significance between groups indicated.

By design, baseline insulin sensitivity (M/I value) was higher in Ob_Sen_ compared with Ob_Res_. M/I was not different between Lean and Ob_Sen_ and was significantly higher in Lean compared with Ob_Res_ (Figure 2A). Similarly, fasting insulin was significantly lower in Ob_Sen_ versus Ob_Res_, and not different between Lean and Ob_Sen_ (Figure 2B).

Circulating adiponectin was significantly higher and FABP4 lower in Lean compared with Ob_Res_ (*p* ≤ 0.026), and concentrations of these adipokines in Ob_Sen_ were not significantly different from either Lean or Ob_Res_. Other circulating cytokines, including FGF19, FGF21, lipocalin-2 and RBP4, were not significantly different between the groups *(p* ≥ 0.23, Table 1).

Self-reported physical activity engagement was not significantly different between the groups (*p* = 0.52, data not shown).

### 3.2. Follow-Up

Average follow-up was 5.4 ± 0.1 years (range 4.3–7.6). Of 99 participants at baseline, 57 participated in the follow-up study (Figure 1). On average, individuals lost to follow-up (for all reasons as detailed in Figure 1, *n* = 42) were younger, significantly leaner and metabolically healthier at baseline, with lower blood pressure and a more favorable baseline lipid profile (Table 2).

BMI remained statistically indistinguishable between Ob_Res_ and Ob_Sen_ at follow-up (*p* = 0.33, Table 1), but, similarly to baseline, visceral fat volume remained lower in Ob_Sen_ compared with Ob_Res_ (*p* = 0.03, Table 1). At follow-up, however, the distinction in liver fat content, measured by ^1^H-MRS, between Ob_Sen_ and the Ob_Res_ group was no longer significant (*p* = 0.31, Table 1).

Between-group analysis at the follow-up time point revealed that M/I at follow-up in Ob_Sen_ remained significantly higher compared to Ob_Res_, and not different from Lean (Figure 2C). Fasting insulin was not significantly different between Ob_Sen_ and Ob_Res_ at follow-up (*p* = 0.13), but remained lower in Lean compared with Ob_Res_ (Figure 2D).

Systolic and diastolic blood pressure remained significantly lower in Ob_Sen_ and Lean compared with Ob_Res_ (*p* ≤ 0.012), but fasting blood glucose was not significantly different between Ob_Res_ and Ob_Sen_ (*p* = 0.37), only significantly lower in Lean versus Ob_Res_ (*p* = 0.004). HDL-cholesterol was significantly higher in Ob_Sen_ compared with Ob_Res_ (*p* = 0.010), and serum triglycerides were significantly lower in Lean compared with Ob_Res_ (*p* = 0.003), but not different between Ob_Sen_ and either Lean or Ob_Res_ (*p* = 0.070, Table 1).

Similarly to baseline, self-reported physical activity engagement was not different between the groups at the follow-up time point (*p* = 0.35, data not shown).

### 3.3. Change in Anthropometry, Metabolic Health and Insulin Resistance

We evaluated the change in metabolic parameters with time, by calculating the difference in the values of the measures from baseline to follow-up, normalising for the time elapsed between the baseline and follow-up studies.

#### 3.3.1. Body Weight and Fat Content

**Figure 3:**
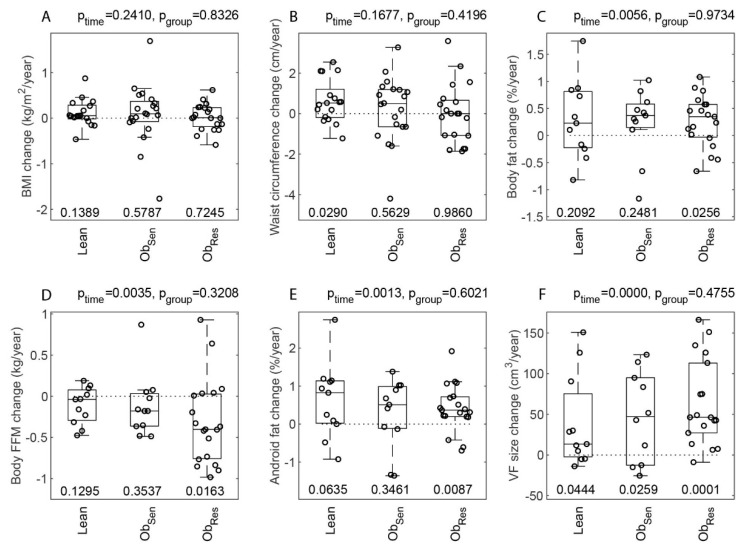

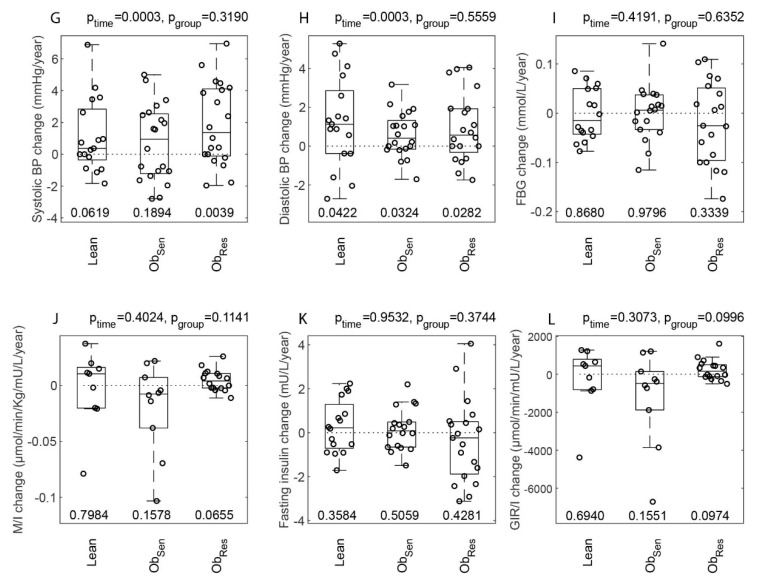
Change in anthropometry, metabolic health and glucose regulation markers from baseline to follow-up in Lean and overweight/obese insulin-resistant and insulin-sensitive individuals. Annual change in body mass index (BMI) (**A**), waist circumference (**B**), body fat (**C**), fat-free mass (**D**), android fat (**E**), visceral fat (**F**), systolic (**G**) and diastolic (**H**) blood pressure, fasting blood glucose (**I**), M/I (**J**), fasting insulin (**K**) and glucose infusion rate (GIR)/I (**L**) in Lean and overweight/obese individuals. Change (IQR) in variables as a function of time, for each of the baseline groups: Lean, Ob_Res_ and Ob_Sen_. The change was determined as $\frac{\text{Follow-up}\;\mathrm{Value}-\mathrm{Baseline}\;\mathrm{Value}}{\mathrm{Time}\;\mathrm{between}\;\mathrm{measurements}}$. Differences between the groups were assessed using a Welsh’s ANOVA (accounting for unequal variances in the change data) with the Games–Howell posthoc test. The p_group_ value is indicated at the top of each plot for each variable. Changes over time for the cohort were assessed using a one-sample *t*-test for difference from zero with *p*_time_ also indicated at the top of each plot. The *p* values for the differences from zero for each individual group are shown at the bottom of each plot. A correction for multiple comparisons (Bonferroni) was applied with significance set at *p* ≤ 0.0167. Data are individual values of change (IQR). Abbreviations: WC, waist circumference; FFM, fat-free mass; SBP, systolic blood pressure; DBP, diastolic blood pressure; FBG, fasting blood glucose.

Surprisingly, BMI (*p*_time_ = 0.24, Figure 3A) and waist circumference (*p*_time_ = 0.17, Figure 3B) did not change significantly over time in this cohort. However, body fat increased significantly over time (*p*_time_ = 0.006, Figure 3C). Other anthropometric and body composition (DXA-derived) variables changed significantly over time, and while none showed a significant difference between groups (Figure 3), in many the significant changes over time were driven by the Ob_Res_ group (as evaluated by one-way *t*-test, Bonferroni-corrected and accepting *p* ≤ 0.0167 as statistically significant). For example, FFM decreased significantly (*p*_time_ = 0.0035, Figure 3D), and the change with time primarily driven by the Ob_Res_ (*p* = 0.0163). Similarly, fat content in the android region increased significantly over time (*p*_time_ = 0.0013, Figure 3E), driven by the Ob_Res_ group (*p* = 0.0087, Figure 3E). Visceral fat volume increased over time (*p*_time_ < 0.001, Figure 3F), driven by a significant increase in the Ob_Res_ (*p* = 0.0001, Figure 3F). On the other hand, gynoid region mass decreased significantly in the cohort (*p*_time_ < 0.001), also driven by a decrease in the Ob_Res_ (*p* = 0.0005).

#### 3.3.2. Liver Fat

Fatty liver status did not change significantly for any of the groups from baseline to follow-up (Chi-squared test *p* = 0.62, *p* = 0.32 and *p* = 1, for Lean, Ob_Sen_ and Ob_Res_, respectively). There was a larger proportion of Ob_Sen_ compared with Ob_Res_ having a stable absence of fatty liver (56 versus 12%); and a larger proportion of Ob_Res_ compared with Ob_Sen_ having a stable presence of fatty liver (76 versus 22%, Table 3).

#### 3.3.3. Cardiovascular Health Markers and Insulin Resistance

Systolic blood pressure increased overall (*p*_time_ = 0.0003, Figure 3G), and significantly in Ob_Res_ (*p* = 0.0039), while diastolic blood pressure, whilst increasing across the whole cohort (*p*_time_ = 0.0003), did not change significantly (with Bonferroni correction) within any of the groups (Figure 3H). Fasting glucose (*p*_time_ = 0.42, Figure 3I), HDL-cholesterol (*p*_time_ = 0.88), LDL-cholesterol (*p*_time_ = 0.75), triglycerides (*p*_time_ = 0.73) and total cholesterol (*p*_time_ = 0.78) did not change significantly over time in this cohort (with little change in the number of individuals treated with lipid-lowering medication, Table 1). Similarly, M/I (*p*_time_ = 0.40, Figure 3J) and fasting insulin (*p*_time_ = 0.95, Figure 3K) did not change significantly over time. As body FFM decreased significantly in Ob_Res_ (Figure 3D, *p*_ObRes_ = 0.0163), we calculated a new variable GIR normalised to circulating steady state insulin, but not normalised to FFM. Similar to M/I, this new variable GIR/I did not change significantly over time in this cohort (*p*_time_ = 0.31, Figure 3L).

### 3.4. Correlates of Follow-Up Insulin Resistance

**Figure 4:**
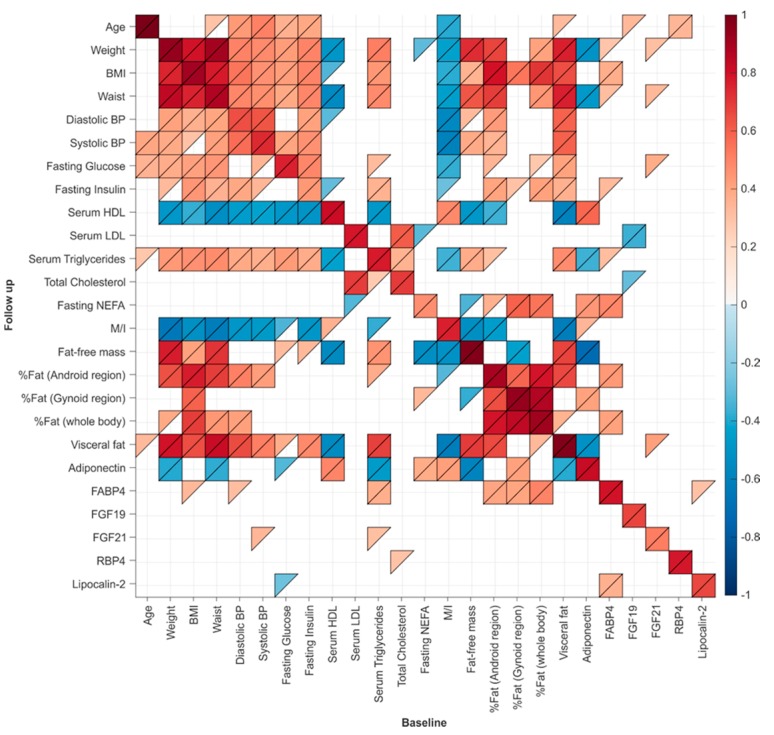
Associations between baseline and follow-up metabolic health, anthropometry and body composition. Pairwise Spearman coefficients were calculated and the *p* values determined. If 0 ≤ *p* < 0.05, then the R value of the correlation is shown in color, with upper triangle indicating 0 ≤ *p* < 0.05, and lower triangle indicating 0 ≤ *p* < 0.01 for the correlation. The correlation coefficient is indicated using the color scale.

Baseline and follow-up anthropometry, metabolic markers and glucose control, including insulin resistance, correlated tightly (Figure 4). The best correlate of follow-up insulin resistance (M/I) was baseline M/I, followed by inverse correlations with central adiposity, measured by visceral fat volume and waist circumference, fat-free mass, BMI, diastolic blood pressure, fasting serum insulin and liver fat (Table 4).

## 4. Discussion

In this study, we followed the change in insulin resistance, metabolic health and body fat composition in individuals with overweight and obesity over a 4.3–7.6 year period. Insulin resistance did not change significantly over time in this cohort, and, similarly to the Lean group, Ob_Sen_ maintained their superior insulin sensitivity relative to Ob_Res_ at follow-up. However, it is important to note that when we retrospectively grouped individuals who were overweight and obese into insulin-sensitive and insulin-resistant sub-groups, the Ob_Sen_ group was significantly younger than the Ob_Res_ group. This is consistent with large cohort epidemiological studies, following the change in metabolic health in individuals who were overweight and obese, reporting that metabolically healthy obesity is rare in older individuals, and that the majority of individuals who are overweight and obese are likely to acquire the metabolic syndrome over time, at ages ranging from early 50s to late 80s [7,8,9,10,11,12,13,14].

Two consistent predictors of maintenance of metabolic health in obesity across previous large cohort longitudinal studies were younger age, and a more peripheral fat distribution [9,15]. In our study, we found that insulin-resistant individuals who were overweight/obese were more susceptible than the insulin-sensitive groups to adverse deposition of abdominal adiposity (android region and visceral fat) and loss of gynoid region and fat-free mass. This was despite no change in BMI or waist circumference over time. Insulin resistance in obesity is strongly associated with muscle mass loss in the elderly, termed “sarcopenic obesity” [25]. In a large cohort of non-obese and obese individuals, Lee and colleagues reported that the proportion of muscle mass (appendicular muscle) to body weight was significantly elevated in men with obesity who remained metabolically healthy over 4 years [26]. The same was found in men and women who were non-obese, but interestingly, not in women with obesity [26]. Skeletal muscle is the primary tissue determining whole body insulin resistance. Fat-free mass loss was observed only in the Ob_Res_ sub-cohort. To our surprise, this was not reciprocated with further deterioration in insulin resistance measured by either hyperinsulinemic-euglycemic clamp or fasting insulin. This may be explained by these methodologies’ lowest detection limits, or by the fact that the FFM loss was not substantial enough to affect whole body insulin resistance.

We and others have suggested that a low degree of liver steatosis is a strong feature of insulin sensitivity in obesity [27]. Indeed, liver fat content measured by CT in Ob_Sen_ in the present study was intermediate, higher compared with Lean and lower compared with Ob_Res_, confirming previous observations [2,28]. Presence or absence of fatty liver over time in the present study did not change for the majority of participants, with more Ob_Sen_ having a stable absence of fatty liver than Ob_Res_ and more Ob_Res_ having a stable presence of fatty liver than Ob_Sen_. At follow-up, the significant distinction in liver fat content between Ob_Sen_ and the Ob_Res_ was lost. We speculate that this relates to the smaller sample size at follow-up, and possibly inadequate statistical power, or the relatively short-term follow-up. Healthy adipose tissue expansion and better capacity to upregulate lipogenesis in adipose tissue [2,29,30] are thought to explain protection against lipid spill-over to the liver and the muscle in insulin-sensitive obesity. While these findings should be interpreted cautiously due to the different modalities used to evaluate liver fat at baseline and follow-up, the maintenance of the baseline liver fat status by the majority of participants is consistent with the lack of significant change in insulin resistance over time in the present study.

Interestingly, the best correlate of future insulin resistance was insulin resistance measured at baseline, suggesting that insulin resistance is an intrinsic characteristic. Other strong predictors were visceral fat, waist circumference, BMI and liver fat. Higher waist circumference [15] and visceral fat area [11] have been previously suggested to predict loss of metabolic health in obesity over time. Here, we complement these findings and maintain that ectopic deposition of fat in the liver at younger age predicts future insulin resistance.

The strengths of our study include the detailed phenotyping using gold-standard measurement of insulin resistance and body fat composition and distribution, and the paired design. A major limitation is the loss of participants to follow-up and the small sample size at follow-up. Furthermore, bias relating to populations likely to volunteer to studies may have affected the findings and may explain the failure to detect changes in body weight over time, contrary to expectations. Lastly, different modalities were used to measure liver fat at baseline and follow-up and the cut-offs used to define fatty liver status were adopted from a different study population, which may have influenced the findings.

## 5. Conclusions

Our findings suggest that insulin resistance at an older age strongly aligns with unfavourable abdominal fat distribution and liver fat measured at a younger age. Furthermore, insulin resistance at a young age predisposes to adverse metabolic outcomes and fat-free mass loss, suggesting that maintenance of body fat-free mass should be encouraged to promote healthy aging in obesity.

## Figures and Tables

**Table 1:** Characteristics of the cohort at baseline and follow-up.

	Baseline				Follow-Up			
Characteristic	Lean	Ob_Sen_	Ob_Res_	ANOVA *p* Value	Lean	Ob_Sen_	Ob_Res_	ANOVA *p* Value
N (M/F)	42 (20/22)	30 (15/15)	27 (12/15)		17 (11/6)	20 (11/9)	20 (8/12)	
Age (years)	45.6 ± 1.9 ^c^	45.2 ± 2.3 ^c^	57.7 ± 2.4 ^a,b^	**0.00 ^1^**	54.6 ± 2.7 ^c^	54.2 ± 2.4 ^c^	63.4 ± 2.4 ^a,b^	**0.01 ^1^**
Weight (kg)	64.4 ± 2.0 ^b,c^	85.1 ± 2.3 ^a^	91.0 ± 2.4 ^a^	**0.00 ^1^**	64.4 ± 3.4 ^b,c^	89.5 ± 3.1 ^a^	92.0 ± 3.1 ^a^	**0.00 ^1^**
BMI (kg/m^2^)	22.2 ± 0.6 ^b,c^	28.9 ± 0.7 ^a^	32.0 ± 0.7 ^a^	**0.00 ^1^**	22.8 ± 1.0 ^b,c^	29.9 ± 0.9 ^a^	32.0 ± 0.9 ^a^	**0.00 ^1^**
Waist circumference (cm)	80 ± 1 ^b,c^	99 ± 2 ^a^	106 ± 2 ^a^	**0.00 ^1^**	83 ± 3 ^b,c^	103 ± 2 ^a^	107 ± 2 ^a^	**0.00 ^1^**
Systolic blood pressure (mmHg)	114 ± 2 ^b,c^	121 ± 2 ^a,c^	134 ± 3 ^a,b^	**0.00 ^1^**	124 ± 4 ^c^	127 ± 4 ^c^	143 ± 4 ^a,b^	**0.00 ^1^**
Diastolic blood pressure (mmHg)	71 ± 1 ^b,c^	76 ± 1 ^a,c^	86 ± 2 ^a,b^	**0.00 ^1^**	79 ± 3 ^c^	80 ± 3 ^c^	91 ± 3 ^a,b^	**0.00 ^1^**
Fasting blood glucose (mmol/L)	4.5 ± 0.1 ^b,c^	4.7 ± 0.1 ^a,c^	5.1 ± 0.1 ^a,b^	**0.00 ^1^**	4.5 ± 0.1 ^c^	4.8 ± 0.1	5.1 ± 0.1 ^a^	**0.00 ^1^**
Total body fat mass (%)	29 ± 1 ^b,c^	38 ± 1 ^a^	41 ± 1 ^a^	**0.00 ^1^**	31 ± 2 ^c^	40 ± 2	42 ± 2 ^a^	**0.00 ^1^**
Android fat mass (% of total android mass)	26 ± 1 ^b,c^	42 ± 1 ^a,c^	47 ± 1	**0.00 ^1^**	29 ± 3 ^b,c^	45 ± 3 ^a^	48 ± 2 ^a^	**0.00 ^1^**
Gynoid fat mass (% of total gynoid mass)	33 ± 1 ^b,c^	39 ± 2 ^a^	41 ± 2	**0.00 ^1^**	34 ± 3 ^c^	41 ± 3	42 ± 2 ^a^	**0.02 ^1^**
Visceral adipose tissue (cm^3^)	358 ± 110 ^b,c^	1123 ± 129 ^a,c^	1962 ± 13 ^a,b^	**0.00 ^1^**	593 ± 293 ^c^	1289 ± 307 ^c^	2370 ± 229 ^a,b^	**0.00 ^1^**
Liver fat *^#^	60 ± 2 ^b,c^	56 ± 2 ^a,c^	43 ± 2 ^a,b^	**0.00 ^1^**	1.4 ± 2.2 ^c^	4.2 ± 2.3	8.6 ± 1.8 ^a^	**0.00 ^1^**
Total cholesterol (mmol/L) ^2^	4.3 ± 0.2	4.8 ± 0.2	4.7 ± 0.2	**0.04**	5.1 ± 0.3	5.1 ± 0.3	4.6 ± 0.2	0.30
Triglycerides (mmol/L) ^2^	0.9± 0.1 ^b,c^	1.3 ± 0.1 ^a^	1.9 ± 0.2 ^a^	**0.00 ^1^**	1.1 ± 0.2 ^c^	1.3 ± 0.2	1.8 ± 0.1 ^a^	**0.00 ^1^**
HDL-cholesterol (mmol/L) ^2^	1.3 ± 0.1	1.3 ± 0.1	1.2 ± 0.1	0.63	1.6 ± 0.1 ^c^	1.4 ± 0.1 ^c^	1.2 ± 0.1 ^a,b^	**0.00 ^1^**
LDL-cholesterol (mmol/L) ^2^	2.6 ± 0.1	2.9 ± 0.2	2.7 ± 0.2	0.15	3.0 ± 0.2	3.1 ± 0.2	2.6 ± 0.2	0.34
Adiponectin (mg/L)	28.6 ± 3.1 ^c^	23.8 ± 2.9	17.4 ± 2.9 ^a^	**0.01 ^1^**	27.7 ± 2.8	23.8 ± 2.7	18.7 ± 2.6	0.07
FABP4 (µg/L)	12.5 ± 5.5 ^c^	18.7 ± 5.0	34.1 ± 4.9 ^a^	**0.01 ^1^**	13.9 ± 6.6	17.0 ± 6.0	32.2 ± 5.7	0.08
FGF19 (ng/L)	149 ± 37	107 ± 33	131 ± 32	0.70	102 ± 26	110 ± 26	112 ± 24	0.96
FGF21 (ng/L)	156 ± 53	143 ± 46	240 ± 40	0.23	151 ± 45	176 ± 35	191 ± 32	0.77
Lipocalin-2 (µg/L)	104 ± 11	93 ± 10	83 ± 10	0.37	114 ± 23	82 ± 22	79 ± 21	0.48
RBP4 (mg/L)	3.0 ± 0.5	2.3 ± 0.5	2.5 ± 0.5	0.60	2.8 ± 0.4	2.6 ± 0.4	2.1 ± 0.4	0.38
Participants treated with anti-hypertensive medication (n)	1	2	8		1	0	4	
Participants treated with lipid lowering medication (n)	0	3	7		1	3	6	

Data are mean ± standard error of the mean (SEM). Significance was tested using one-way Analysis of Variance (significant values highlighted in bold) with Games–Howell post hoc analyses. ^a^ mean is significantly different to Lean, *p* < 0.05; ^b^ mean is significantly different to Ob_Sen_, *p* < 0.05; ^c^ mean is significantly different to Ob_Res_, *p* < 0.05. ^1^ Where the homogeneity of variances assumption was violated, the Welch statistic was used with Games–Howell post hoc. * Baseline based on CT attenuation (Hu) and follow-up based on ^1^H-MRS (%). ^#^ Liver attenuation by CT is an inverse correlate of liver fat content. ^2^ Data logged prior to statistical analyses. Abbreviations: FGF, fibroblast growth factor; FABP4, fatty-acid binding protein 4; RBP4, retinol-binding protein 4.

**Table 2:** Baseline characteristics of participants who were lost to follow-up versus those who were studied.

Baseline Characteristic	Not Studied	Studied	*p* Value
N (M/F)	42 (20/22)	57 (27/30)	**0.00 ^1^**
Age (years)	44.4 ± 2.0	52.1 ± 1.7	**0.00 ^1^**
BMI (kg/m^2^)	25.2 ± 0.8	28.1 ± 0.7	**0.00 ^1^**
Waist circumference (cm)	88 ± 2	96 ± 2	**0.00 ^1^**
Systolic blood pressure (mmHg)	117 ± 2	125 ± 2	**0.01 ^1^**
Diastolic blood pressure (mmHg)	75 ± 2	78 ± 1	**0.04 ^1^**
Fasting blood glucose (mmol/L)	4.5 ± 0.1	4.8 ± 0.1	**0.00 ^1^**
Fasting insulin (mU/L)	11.7 ± 1.1	14.4 ± 0.9	**0.03 ^1^**
Total cholesterol (mmol/L) ^2^	4.1 ± 0.2	4.9 ± 0.1	**0.00 ^1^**
Triglycerides (mmol/L) ^2^	1.1 ± 0.1	1.5 ± 0.1	**0.01 ^1^**
HDL-cholesterol (mmol/L) ^2^	1.1 ± 0.1	1.4 ± 0.0	**0.00 ^1^**
LDL-cholesterol (mmol/L) ^2^	2.5 ± 0.1	2.9 ± 0.1	**0.02 ^1^**

Data are mean ± SEM. ^1^ Where the homogeneity of variances assumption was violated, the Welch statistic was used. Significance was tested using one-way Analysis of Variance (significant values highlighted in bold). ^2^ Data logged prior to statistical analyses. Abbreviations: HDL: high-density lipoprotein, LDL: low-density lipoprotein.

**Table 3:** Presence of fatty liver at baseline and follow-up in Lean, Ob_Sen_ and Ob_Res_.

Presence of Fatty Liver (Baseline to Follow-Up) *	Study Group		
	Lean (*n*, % of Group)	Ob_Sen_ (*n*, % of Group)	Ob_Res_ (*n*, % of Group)
Stable Absence (*n* = 14)	7 (64%)	5 (56%)	2 (12%)
Gain of fatty liver (*n* = 5)	2 (18%)	2 (22%)	1 (6%)
Loss of fatty liver (*n* = 2)	1 (9%)	0 (0%)	1 (6%)
Stable Presence (*n* = 16)	1 (9%)	2 (22%)	13 (76%)
Total (*n* = 37)	11 (100%)	9 (100%)	17 (100%)

Paired liver fat measurements were available for a sub-cohort of 37 individuals who had CT and MRS at baseline and follow-up, respectively. * Presence of fatty liver determined by a CT value of <54.2 Hu at baseline and MRS > 1.8% at follow-up, as described by van Werven et al. [21]. Terminology: Stable Absence: Fatty liver absent at baseline and follow-up; Gain of fatty liver: Fatty liver absent at baseline but present at follow-up; Loss of fatty liver: Fatty liver present at baseline but absent at follow-up; Stable Presence: Fatty liver present at baseline and follow-up.

**Table 4:** Baseline correlates of follow-up insulin resistance (M/I).

Baseline Measure	R	*p*
M/I	0.76	**1.1 × 10^−07^**
Visceral fat volume	−0.63	**5.7 × 10^−05^**
Waist circumference	−0.63	**5.8 × 10^−05^**
Fat-free mass	−0.53	**0.001**
BMI	−0.52	**0.001**
Diastolic blood pressure	−0.49	**0.003**
Fasting Insulin	−0.50	**0.003**
Liver attenuation (Hu) *	0.49	**0.003**
Systolic blood pressure	−0.47	**0.004**
Fat in the android region (% of total abdominal fat)	−0.46	**0.006**
Serum triglycerides	−0.38	**0.025**
Serum HDL cholesterol	0.36	**0.034**
Adiponectin	0.34	**0.044**
Fasting blood glucose	−0.34	**0.046**
Age	−0.33	0.052
FGF21	−0.29	0.126
FABP4	−0.26	0.149
Body fat (% whole body mass)	−0.25	0.154
Serum LDL	0.24	0.163
Total Cholesterol	0.19	0.277
RBP4	0.06	0.711
Fasting NEFA	0.07	0.714
Lipocalin−2	0.05	0.764
FGF19	−0.03	0.878
Fat in the gynoid region (% of total abdominal fat)	−0.02	0.922

Spearman coefficients were calculated. Significant *p* values are bolded; * Liver attenuation by CT is an inverse correlate of liver fat content.
